# Approaches for completing metabolic networks through metabolite damage and repair discovery

**DOI:** 10.1016/j.coisb.2021.100379

**Published:** 2021-12

**Authors:** Corey M. Griffith, Adhish S. Walvekar, Carole L. Linster

**Affiliations:** Luxembourg Centre for Systems Biomedicine, University of Luxembourg, Esch-sur-Alzette, Luxembourg

**Keywords:** Metabolite repair enzymes, Underground metabolism, Non-canonical metabolites, Untargeted metabolomics

## Abstract

Metabolites are prone to damage, either via enzymatic side reactions, which collectively form the underground metabolism, or via spontaneous chemical reactions. The resulting non-canonical metabolites that can be toxic, are mended by dedicated “metabolite repair enzymes.” Deficiencies in the latter can cause severe disease in humans, whereas inclusion of repair enzymes in metabolically engineered systems can improve the production yield of value-added chemicals. The metabolite damage and repair loops are typically not yet included in metabolic reconstructions and it is likely that many remain to be discovered. Here, we review strategies and associated challenges for unveiling non-canonical metabolites and metabolite repair enzymes, including systematic approaches based on high-resolution mass spectrometry, metabolome-wide side-activity prediction, as well as high-throughput substrate and phenotypic screens.

## Highlights

• Underground metabolism can generate useful, useless, or toxic metabolites.
• Damaged metabolites can be produced non-enzymatically or owing to enzyme promiscuity.
• Metabolite repair enzymes eliminate damaged metabolites or pre-empt their formation.
• Tools to discover underground pathways could be used to reveal hidden repair systems.
• Metabolite repair is crucial to account for in disease and metabolic engineering.

## Graphical abstract

**Figure:**
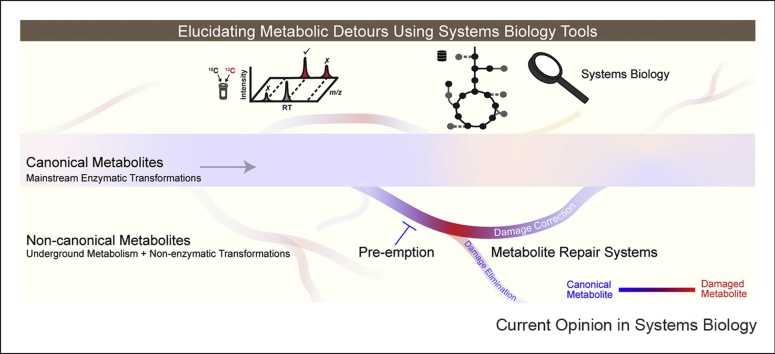

## Glossary

Non-canonical metabolites: small molecules formed from intracellular metabolites by enzymatic side reactions or non-enzymatic reactions; they are typically not intermediates in any metabolic pathway.
Substrate promiscuity: property of an enzyme to utilize multiple substrates.
Catalytic promiscuity: property of an enzyme to catalyze more than one type of chemical reaction on a given substrate.
Underground metabolism: often neglected part of cellular metabolism encompassing the side-reactions catalyzed by metabolic enzymes on endogenous substrate analogs.
Damaged metabolites: toxic or useless non-canonical metabolites.
Metabolite repair: enzymatic transformation of a damaged metabolite back to the canonical precursor metabolite. The term “metabolite repair enzyme” can also designate enzymes that eliminate damaged metabolites or pre-empt their formation. Such enzymes are also referred to as metabolite damage-control, house-cleaning, or proofreading enzymes in literature.
Metabolic engineering: process involving genome editing and/or (heterologous) pathway/enzyme (over)expression in host organisms, with the aim to produce novel metabolites/value-added chemicals.
Credentialing: used to describe analytical methods that allow identification of biologically-derived peaks in mass spectrometry-based metabolomics data generated from unlabeled and stable isotope labeled extracts.
Protein moonlighting: expression used to describe proteins that have more than one physiologically relevant function (e.g., catalytic activity and transcriptional regulation).

## Introduction

Historically, metabolic enzymes were thought to drive linear pathways by catalyzing successive, specific transformations; however, we now know that non-canonical metabolites are generated concurrently. This is due to the lack of perfect substrate and/or reaction specificity of metabolic enzymes, as well as inherent reactivity and instability of certain metabolites [1, 2, 3, 4]. Enzyme promiscuity feeds the underground metabolic network, where enzymes act on endogenous substrate analogs and thereby increase the chemical diversity of the intracellular metabolite pool [5]. While ‘unintended’ enzymatic or non-enzymatic transformations most often yield useless or toxic metabolites, enzymatic side activities can confer adaptive advantages under changing environmental conditions for example [6, 7, 8•]. Toxic metabolic side products call for dedicated “metabolite repair enzymes” (also designated metabolite damage-control, proofreading or housecleaning enzymes) to pre-empt their formation, convert them to harmless products, or reconvert them to benign precursor substrates (for comprehensive overviews describing previously discovered repair enzymes, we refer the reader to a number of excellent reviews [9, 10, 11, 12, 13]). The reciprocal examples where the side-activity of an enzyme clears damaged metabolites are rare [14,15], suggesting that repair enzymes face stronger evolutionarily selection pressures to retain the repair activity as their primary function.

Underground metabolism and metabolite repair are conceptually linked via the notion of enzyme promiscuity and overlap where the former produces toxic or wasteful metabolites (Supplementary Table S1) that need to be cleared or recycled by the latter. Apart from inherited metabolic disorders [16,17], underground metabolism and metabolite repair have implications in metabolic engineering where metabolic rewiring and enzyme overexpression can increase the production of non-canonical metabolites and integration of metabolite repair systems can increase fitness of engineered systems [18]. Recent studies seek to identify underground metabolic detours using computational tools [19,20] and exploit promiscuous enzymes as entry points for conversion of inexpensive chemicals to value-added ones [7,21].

The apparent paradox between the dwindling number of remaining gaps in primary metabolic pathways and the high number of remaining enzymes of unknown function [22], the fact that most metabolic enzymes catalyze side-reactions, and the observation that the peaks detected by untargeted metabolomics largely outnumber the metabolites contained in metabolic reconstructions [23,24], indicate that many more metabolite repair enzymes remain to be identified. Growing realization that metabolite repair systems can act as a targetable liability in diseases [25], benefit metabolic engineering endeavors [18], and provide fitness advantages under conditions of stress [26], emphasizes the relevance of continuing to unveil hidden repair systems. Here, we review and propose strategies that have been used or could aid in discovering non-canonical metabolites and metabolite repair enzymes, focusing on most recent studies for illustration and emphasizing the important role played by systems biology approaches in this type of research.

## Non-canonical metabolite discovery

**Figure 1:**
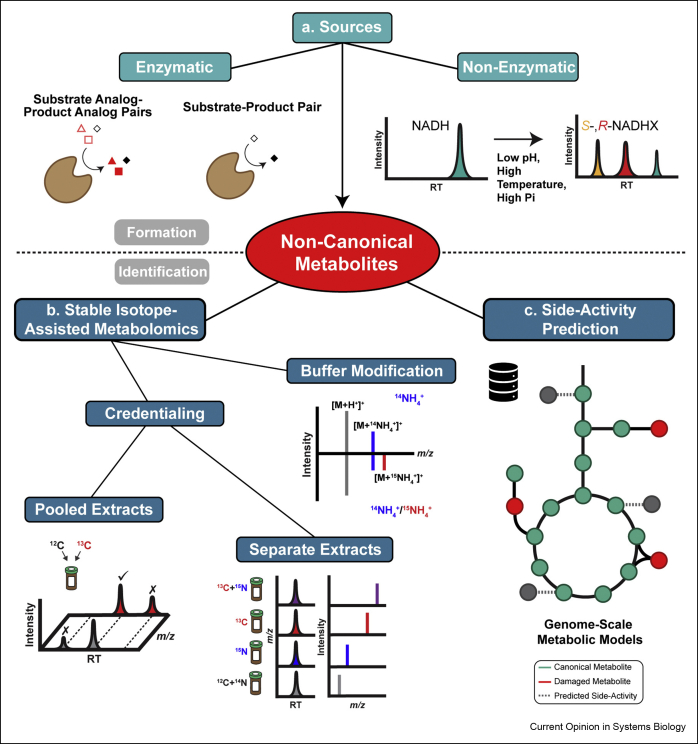
Sources and discovery strategies for non-canonical metabolites. **a**) Non-canonical metabolites are formed via enzymatic side reactions and non-enzymatic reactions, such as spontaneous hydration of NADH to *S*- and *R*-NADHX. **b**) Stable-isotope assisted metabolomics approaches hold great promise for further identification of non-canonical metabolites. Pooled extracts of unlabeled and labeled samples can be combined prior to analysis, allowing for identification of credentialed (i.e., biologically-derived) features in a single analysis. Using separate extracts, credentialed features are aligned and identified post acquisition, with *m/z* shifts corresponding to the number of labeled atoms. The Buffer Modification Workflow identifies adducts formed with unlabeled mobile phase eluents (top spectrum) and partially labeled eluents (bottom spectrum), as observed in the proportional decrease in ^14^NH_4_^+^ adduct and increase in ^15^NH_4_^+^ adduct depicted in the example shown. **c**) Combining genome-scale metabolic models and cheminformatic tools offers strategies for more systematic predictions of metabolic network expansions (side-activities) which can facilitate non-canonical metabolite annotation and identification.

Important technological advances in gas chromatography (GC) and liquid chromatography (LC) coupled with high-resolution mass spectrometry (HRMS) are driving the development of more and more sensitive, rapid, and comprehensive methods to analyze the cellular metabolome, including the products of underground metabolism. While untargeted metabolomic analyses generate datasets of increasing size and complexity, related data analysis method development is lagging behind. Metabolite annotation remains challenging due notably to the inability to differentiate, among the 10,000s features (i.e., peaks) detected in a single run, biologically relevant features from features that are not (or not directly) derived from the analyzed biological systems (e.g., adducts, in-source fragments, environmental contaminants). Cheminformatic and other computational methods are needed to find and align peaks, perform MS/MS spectral matching, and aid unknown peak identification. Discovering non-canonical metabolites in untargeted datasets is even more challenging since their intracellular concentration is typically maintained at low levels by repair enzymes. Additionally, their chemical structures are highly diverse and may be unexpected since they arise from enzyme promiscuity and non-enzymatic reactions of canonical metabolites (Figure 1a). Even when detected, predicting relevant enzymatic side-activities or spontaneous byproducts is not usually incorporated in computational metabolomic workflows, which thus need to be adapted for increased capture of non-canonical metabolites.

### Stable isotope-assisted metabolomics

*Credentialing* (Figure 1b) is a stable isotope-assisted technique that strives to distinguish biologically-derived features from environmental noise in untargeted mass spectrometry metabolomics data to reduce data complexity and facilitate annotation. Typically, cell extracts are prepared after cultivation in media supplemented with unlabeled and/or labeled (e.g., ^13^C, ^15^N) substrates. By comparing the data derived from the unlabeled and stable isotope-labeled samples, credentialing software detects and retains features with identical retention times and a mass shift corresponding to the number of labeled atoms (Figure 1b), reducing feature counts from 10,000s to less than 1000s [27,28]. Credentialing is appealing for non-canonical metabolite discovery because it assigns biological relevance to overlooked peaks in untargeted data. Credentialing software (e.g., X^13^CMS, mzMatch-ISO, geoRge, MetExtractII, PAVE, MSDial) is applied to data generated from *pooled* or *separate extracts* (Figure 1b) of labeled and unlabeled samples [29, 30, 31, 32, 33, 34•, 35]. The use of pooled extracts saves time and simplifies data processing since labeled and unlabeled compounds co-elute; however, this approach dilutes samples. The latter is avoided when analyzing separate extracts, but here retention time shifts can complicate data analysis.

The Peak Annotation and Verification Engine (PAVE) was developed for credentialing in separate extracts (Figure 1b) and, in a proof-of-concept study using *Saccharomyces cerevisiae* and *Escherichia coli* extracts, retained approximately 2000 features as apparent metabolites (i.e., 4% of all the peaks detected) [34]. Of these, 220 features matched with authenticated standards based on MS/MS and/or retention time. The remaining credentialed metabolites provide a manageable list of biologically-derived features worth annotating, potentially including novel and non-canonical metabolites. In an effort to define the active metabolome of the malaria parasite *Plasmodium falciparum*, erythrocytes infected with the parasite were credentialed using ten ^13^C-tracer substrates (e.g., ^13^C-glucose, ^13^C-amino acids) and subjected to untargeted MS-based metabolomic analyses [36]. The 911 identified metabolites covered 41% of the metabolome predicted by the metabolic reconstruction of infected erythrocytes. Interestingly, 89 observed metabolites were not predicted by the metabolic reconstruction, with many corresponding to damaged metabolites (e.g., 2-hydroxyglutarate, 4-phosphoerythronate, 2-phospholactate). Although elucidation of novel non-canonical metabolites was not a focus of the study, it highlights the utility of credentialing for non-canonical metabolite annotation.

A major limitation with credentialing is that not all models or samples are amenable to isotope labeling (e.g., animals). Here, the LC-MS-based *Buffer Modification* Workflow (BMW) (Figure 1b) can be used as a prioritization strategy that identifies buffer-derived adduct species, formed notably during electrospray ionization, in untargeted data [37]. BMW is based on the use of both unlabeled and partially labeled eluent buffers. It does not allow for credentialing, but simplifies annotation by reducing spectral complexity. Lastly, derivatization can additionally be employed to stabilize reactive non-canonical metabolites (e.g., methylglyoxal) [2,4]. This is also appealing in the frame of LC-MS analyses, since derivatization typically increases metabolite hydrophobicity, improving ionization and sensitivity owing to elution in higher organic phase percentages [38,39]. Derivatization with unlabeled or stable isotope-labeled reagents could be combined with credentialing software to identify derivatized features (not limited to metabolites), providing another method to prioritize relevant features in untargeted metabolomics data.

### Side-activity prediction

Genome-scale metabolic models (GEMs), although incomplete because of unknown enzymes and reactions [40,41], provide an organism-specific template for predicting products of enzyme promiscuity (Figure 1c) using tools such as Metabolic *In silico* Network Expansions (MINEs), the “Enzyme Commission-based” option of BioTransformer, or Extended Metabolic Models (EMM) [42, 43, 44]. Enzymatic side-activity predictions allow for the assembly of suspect lists that can be screened for in untargeted metabolomics data. For instance, the MINEs database was used to annotate 8 unknown features detected by untargeted GC-HRMS, including 1-dehydro-1-deoxy-glucose-6-phosphate, potentially formed by phosphorylation of a non-canonical sugar, in human cancer cells [45]. Similarly, promiscuity prediction using EMM helped confirm the presence of 4-hydroxyphenyllactate, a non-canonical tyrosine metabolite, in CHO cells [46].

GEM-PROPER combines the *E. coli* GEM and unsupervised PSI-BLAST to predict promiscuous replacer genes that could compensate for deficient essential metabolic genes [19]. Thiazole synthase (*thiG*) was for example predicted (and subsequently validated) as an indirect replacer enzyme for erythronate-4-phosphate dehydrogenase (*pdxB*) in the *E. coli* pyridoxal 5′-phosphate (PLP) biosynthesis pathway. The Metabolic Disruption Workflow (MDflow) combines GEMs and EMM promiscuity prediction to evaluate the impact of heterologous enzyme expression or gene suppression/overexpression on metabolism [20]. Inclusion of a toxicity index in workflows such as MDflow could indicate hotspots where repair enzymes may be required; such inclusions would be valuable for metabolite repair discovery as well as for designing robust, modular metabolic engineering models.

## Metabolite repair enzyme discovery

**Figure 2:**
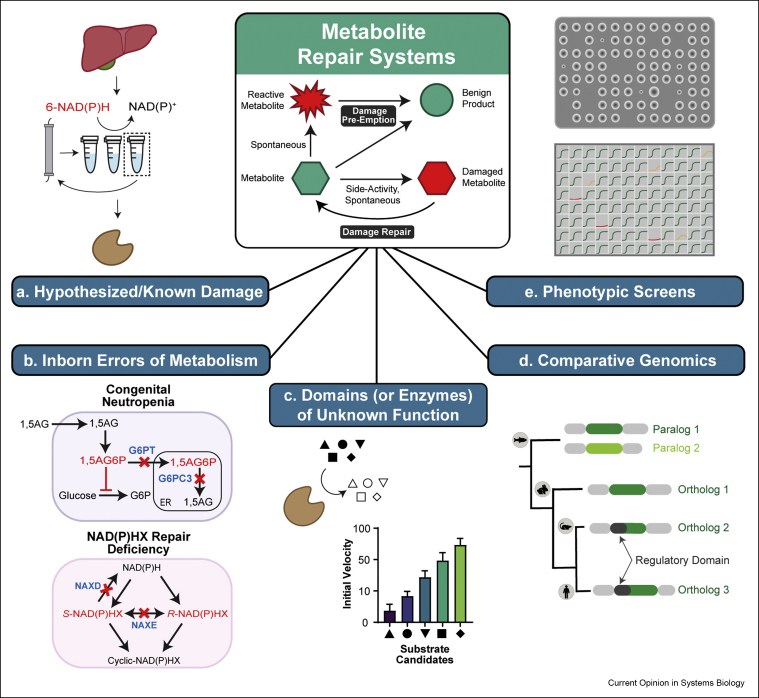
Discovery strategies for metabolite repair systems. **a**) Purification of a putative repair enzyme from tissues (e.g., liver) based on a specific enzymatic assay followed by protein sequence identification by tandem MS. **b**) Building on the knowledge of non-canonical metabolites (e.g., 1,5AG6P, NAD(P)HX) accumulating in inborn errors of metabolism and/or the underlying gene defects (e.g., *G6PT*, *G6PC3, NAXD*, *NAXE*). 1,5AG, 1,5-anhydroglucitol; 1,5AG6P, 1,5-anhydroglucitol-6-phosphate; G6P, glucose-6-phosphate; G6PT, glucose-6-phosphate transporter; G6PC3, glucose-6-phosphate catalytic subunit 3; ER, endoplasmic reticulum; NAXD, NAD(P)HX dehydratase; NAXE, NAD(P)HX epimerase. **c**) *In vitro* substrate screens with recombinant enzymes of unknown function. **d**) Using comparative genomics to find new repair activities in other species (e.g., after gene duplication) or through conserved genome clustering with promiscuous enzymes (especially in prokaryotic operons; not shown). **e**) Phenotypic screenings of genetic models (deficient in repair enzyme candidates) using for example high-throughput growth assays on solid (top) or in liquid (bottom) media. Green, normal growth; yellow, impaired growth; red, no growth.

It is likely that a substantial fraction of remaining enzymes of unknown function are involved in metabolite damage-control [22]. Metabolite repair enzyme discovery relies on biochemical and analytical methods to characterize purified native or recombinant enzymes and demonstrate damage accumulation in repair-deficient cell or whole organism models (Figure 2). Relevant models studied in adequate conditions are crucial for progressing in our understanding of the physiological role of metabolite repair systems. Damage accumulation can be growth phase-dependent [47] and/or diluted by rapid cell division [48]. Slow-growing or post mitotic cells may therefore be less resilient to metabolite repair deficiencies, as observed in related human disorders causing neurodegeneration or neutropenia ([49, 50, 51, 52••, 53] and see below). This highlights the relevance of using cell-type or tissue-specific and whole organism models in metabolite repair research. Figure 2 provides an overview of starting points and strategies used for discovering metabolite repair enzymes and pathways.

*Hypothesized or known metabolite damages* (Figure 2a) can be a good starting point for repair enzyme identification as illustrated by a number of past discoveries [54, 55, 56]. The known damaged metabolites 6-NAD(P)H (differing from the canonical cofactors by the position of the reduced carbon in the nicotinamide ring) had originally been shown to be oxidized to normal NAD(P)^+^ by renalase, an enzyme that is highly expressed in kidney and heart. More recently, 6-NAD(P)H were found to be actively degraded also in rat liver extracts [15]. Protein fractionation of the latter unexpectedly revealed a side activity of pyridoxamine-phosphate oxidase (PNPO) to be responsible for this repair activity. This indicates that in mammals 6-NAD(P)H accumulation is prevented by renalase in heart and kidney and by PNPO in liver and potentially other tissues where renalase is not expressed. Interestingly, based on these findings, PNPO-related enzymes have been identified in other species that are not active on pyridoxamine-phosphate, but seem to have conserved the repair activity as their primary function [15].

*Inborn errors of metabolism* (Figure 2b) can reveal damaged metabolites and lead to repair enzyme discovery as first demonstrated by the now classical example of L-2-hydroxyglutaric aciduria [49]. More recently, the molecular basis of another inborn error of metabolism characterized by severe congenital neutropenia was elucidated via discovery of the metabolite repair function of the deficient enzyme. G6PC3, a glucose-6-phosphatase homolog thought to be involved in glucose-6-phosphate metabolism, was shown to collaborate with G6PT (glucose-6-phosphate transporter) to degrade the non-canonical metabolite 1,5-anhydroglucitol-6-phosphate (1,5AG6P) [52]. The latter is formed by promiscuous phosphorylation of 1,5-anhydroglucitol (1,5AG), a polyol commonly found in food, by cytosolic ADP-glucokinase and low-*K*_m_ hexokinases. G6PT transports 1,5AG6P into the endoplasmic reticulum where it can then be dephosphorylated by G6PC3. 1,5AG6P accumulation inhibits hexokinases and decreases flux towards metabolic pathways that are critical for neutrophils by depleting the glucose-6-phosphate pool. Based on these insights, the antidiabetic drug empagliflozin was repurposed to successfully treat this neutropenia by enhancing urinary excretion of 1,5AG and thereby lowering its levels in blood [57]. Inability to repair damaged (hydrated) NAD(P)H (designated NAD(P)HX) leads to a severe infantile neurodegenerative disorder which can be caused by loss-of-function mutations in either of the NAD(P)HX repair enzymes NAXD (dehydratase) or NAXE (epimerase) [50,51]. Here, the repair enzymes had been discovered before the associated human disease [56], based on very early *in vitro* studies that had demonstrated the promiscuous formation of NADHX by the glycolytic enzyme GAPDH [58].

*Domains (or enzymes) of unknown function* (*DUFs;* Figure 2c) represent another starting point for the search, often challenging, of metabolite repair enzymes. In 2016, Huang et al. identified members of the DUF89 protein family as metal-dependent phosphatases with potential metabolite repair roles [59]. The *S. cerevisiae* DUF89 protein Ymr027w showed highest activity on fructose-1-phosphate, a glycating agent and non-canonical metabolite in yeast that accumulated in *YMR027W* deletion strains*.* An *in vitro* phosphatase screen against an array of phosphoesters with ARMT1, the human homolog of Ymr027w [60], also showed highest activity with fructose-1-phosphate, but deficient cell models were not analyzed in this study for further validations*.* Other members of the DUF89 family, including human *PANK4*, hydrolyze non-canonical oxidized forms of 4′-phosphopantetheine [59]. Interestingly, a causal link was recently identified between a mutation in *PANK4* and a congenital cataract form [61].

*Comparative genomics* (Figure 2d) is fruitful for unveiling new repair enzyme gene candidates since the damage-control part of primary metabolism is often well-conserved across species. Sequence comparisons suggested that the *P. falciparum* gene *PF3D7_0715000* encodes phosphoglycolate phosphatase (PGP) [25], a metabolite repair enzyme for 4-phosphoerythronate (4 PE) and 2-phospholactate (2 PL), glycolytic side-products that inhibit the pentose phosphate pathway (PPP) and glycolysis, respectively [62]. *P. falciparum* Δ*pgp* mutants indeed showed 4 PE and 2 PL accumulation, reduced glycolytic flux and interestingly, increased sensitivity to fosmidomycin, an anti-malaria drug, providing the first indication of a metabolite repair enzyme as a drug target candidate. RidA is a widely conserved damage pre-empting deaminase acting on reactive enamines/imines (e.g., 2-aminoacrylate), which inhibit PLP-dependent enzymes. While RidA is encoded by a single-copy gene in most eukaryotes, teleost fish genomes contain two paralogs of RidA [63]. Imine analog screenings showed that *Salmo salar* RidA-1 had greater activity on 2-iminoacids derived from nonpolar amino acids, like the mammalian homolog, while RidA-2 showed higher activity on 2-iminoacids derived from glutamate and aromatic amino acids. Since gene clustering in bacterial genomes can hint at functional relationships, a particularly fruitful comparative genomics approach consists of searching bacterial chromosomes for genes clustering in a conserved manner with enzymes known to produce damaged metabolites; such genes should be considered as metabolite repair gene candidates. In this way, using the SEED database and its tools [64], Niehaus et al. recently identified a new prokaryotic repair enzyme that prevents accumulation of the damaged metabolite 5-oxoproline [65].

*Phenotypi*c *screens* (Figure 2e) in which mutant strains are systematically exposed to an array of growth media (e.g., supplemented with side-activity precursors) or environmental conditions (e.g., temperature, pH, osmotic stress) can “unmask” phenotypes. This is notably true for metabolite repair genes, which are typically not essential and even phenotypically silent under standard conditions. Growth phenotyping of a *Bacillus subtilis* strain deleted in the ribosome assembly GTPase CpgA unexpectedly revealed a high sensitivity to exposure to carbon sources feeding into the PPP (glucose, gluconate and ribose) [66]. The observed growth defects were finally linked to accumulation, in the Δ*cpgA* mutant, of the damaged metabolite and PPP inhibitor 4 PE (see above). While 4 PE accumulation is prevented in other species by a dedicated repair phosphatase (PGP [25,62]), this role thus seems to be covered by a moonlighting function of CpgA in *B. subtilis*. Finally (pooled) loss-of-function screens (e.g., using barcoded CRISPRi or RNAi libraries), can be designed to identify new enzyme functions [67]. Although not an example of metabolite repair, this approach is illustrated by a recent study where a CRISPRi phosphatase knockdown library was devised to identify promiscuous phosphatases interfering with the *E. coli* terpenoid biosynthesis pathway [68]. Competition assays using pools of barcoded strains knocked out or down for unknown or suspected repair enzymes under an array of unmasking conditions could be used to identify genes that confer subtle growth advantages [69,70]. In higher organisms, CRISPRi knockdown libraries of unknown enzymes more highly expressed in post mitotic tissues could be developed to prioritize metabolite repair enzyme candidates for further investigation [71].

## Leveraging systems biology approaches to uncover distal effects of metabolite repair deficiencies

**Figure 3:**
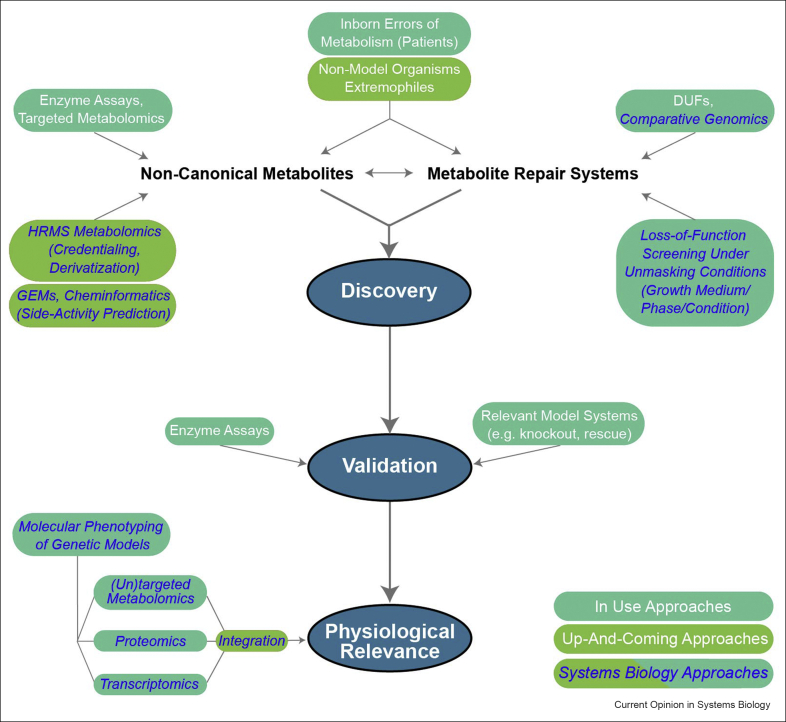
Current and potential approaches for metabolite damage and repair research. Overview of current (dark green boxes) and up-and-coming (light green boxes) approaches for discovery, validation, and elucidation of physiological relevance of metabolite damage and repair systems. Systems biology approaches are marked with italicized blue font. HRMS, high-resolution mass spectrometry; DUFs, domains of unknown function; GEMs, genome-scale metabolic models.

As illustrated through various examples above, inborn errors of metabolism and classical biochemistry approaches have largely contributed to original metabolite repair enzyme discoveries (Figure 3). The relatively younger systems biology approaches have also played an important role in elucidating underground metabolism and, as described above, hold great promise in accelerating prediction and identification of non-canonical (including damaged) metabolites (Figure 3) [20,34,37,42, 43, 44].

Omics approaches provide a means to unravel the systems-wide (distal) impacts of metabolite damage accumulation (which represents the proximal effect of metabolite repair deficiencies), often beyond what can be predicted and have already contributed to understanding the physiological relevance of selected repair systems. Transcriptomic and metabolic profiling of the *Salmonella enterica* Δ*ridA* strain showed the anticipated 2-aminoacrylate accumulation and inhibition of PLP-dependent branched-chain amino acid and folate metabolism, but unexpectedly revealed alterations in nucleotide and SAM-dependent metabolism [72]. Transcriptomic and metabolic profiling of a *S. cerevisiae* NAD(P)HX dehydratase knockout strain uncovered perturbed serine metabolism, which was then shown to result from inhibition by NADHX of the serine synthesis pathway at the level of 3-phosphoglycerate oxidation [47]. Comparative genomics and metabolic profiling of *E. coli* NAD(P)HX epimerase mutants suggested a moonlighting function of this repair enzyme in PLP metabolism [73]. Furthermore, proteomic analyses indicated perturbations in motility, glycolysis, and tricarboxylic acid cycle in a *B. subtilis* NAD(P)HX dehydratase knockout strain under osmotic and ethanol stress [74]. Lastly, untargeted GC- and LC-MS approaches combined with deuterium labeling were employed to identify non-canonical branched-chain fatty acids accumulating in adipocytes deficient in ECHDC1 [75], a repair enzyme acting on (m)ethylmalonyl-CoA [55] and recently linked to certain cases of ethylmalonic aciduria [53]. These studies highlight the relevance and opportunities for (multi-)omics approaches (and their integration) in investigating the global impact of repair deficiencies on cell function, and metabolism in particular (Figure 3). Applying genetic and multi-omic approaches in non-model organisms and extremophiles could further identify niche-specific metabolite damages and their repair systems.

## Conclusions

The concepts of underground metabolism and metabolite repair have breathed new life into metabolic research, inspiring the discovery of new metabolites, enzymes, and entire pathways, demystifying poorly understood diseases to the point of making them treatable, and adding new parts to the toolbox of metabolic engineers. Comprehensive molecular phenotyping techniques have provided invaluable insights into the functional consequences of repair deficiencies, but the next opportunity for advancing this young field lies in a better utilization of systems biology tools to continue pulling underground and repair metabolism out of the shadows *en route* to completing metabolic networks (Figure 3).

## Conflict of interest statement

None declared.
