# Supplementary material for: Approaches for completing metabolic networks through metabolite damage and repair discovery
Source: Curr Opin Syst Biol. 2021 Dec;28:None. doi: 10.1016/j.coisb.2021.100379 (PMC8669784; doi:10.1016/j.coisb.2021.100379)
Supplement: Multimedia component 1 [file mmc1.doc]

**Supplementary Table S1: Damaged** metabolites produced by underground metabolism.

| **Damaged/Toxic metabolite** | **Enzyme (side-activity produces the damaged metabolite)** | **Reference** | **Comment** |
| --- | --- | --- | --- |
| NAD(P)HX | Glyceraldehyde-phosphate dehydrogenase | [1] |  |
| 5-Formyltetrahydrofolate | Serine hydroxymethyltransferase | [2] |  |
| L-2-Hydroxyglutarate | L-Malate dehydrogenase | [3] |  |
| D-2-Hydroxyglutarate | 3-Phosphoglycerate dehydrogenase  Isocitrate dehydrogenase | [4]  [5] |  |
| Ethylmalonyl-CoA | Acetyl-CoA carboxylase | [6] |  |
| L-Pipecolate | Pyrroline-5-carboxylate reductase | [7] |  |
| GDP-D-glucose | GDP-D-mannose pyrophosphorylase | [8] |  |
| Dinucleoside polyphosphates (particularly Ap4A, diadenosine tetraphosphate) | Various enzymes that have acyl-adenylate and/or enzyme-adenylate intermediate | [9] |  |
| 1,5-Anhydroglucitol-6-phosphate | ADP-glucokinase and low-KM hexokinases | [10] |  |
| Methylglyoxal | Triose phosphate isomerase | [11] | Although largely portrayed as a toxic metabolite, the exact role of methylglyoxal under physiological conditions is still not clear. |
| 4-Phosphoerythronate | Glyceraldehyde-3-phosphate dehydrogenase | [12] | All are dephosphorylated by the repair enzyme, phosphoglycolate phosphatase [13] |
| 2-Phospho-L-lactate | Pyruvate kinase | [14] |
| 2-Phosphoglycolate | Pyruvate kinase, Rubisco | [15–17] |
| Deaminated glutathione | Various transaminases | [18] |  |
| Oxalate | L-Lactate dehydrogenase | [19] |  |

**References:**

1. Rafter GW, Chaykin S, Krebs EG: **The action of glyceraldehyde-3-phosphate dehydrogenase on reduced diphosphopyridine nucleotide.** *J Biol Chem* 1954, **208**:799–811.

2. Stover P, Schirch V: **Serine hydroxymethyltransferase catalyzes the hydrolysis of 5,10-methenyltetrahydrofolate to 5-formyltetrahydrofolate.** *J Biol Chem* 1990, **265**:14227–14233.

3. Rzem R, Vincent M-F, Van Schaftingen E, Veiga-da-Cunha M: **L-2-hydroxyglutaric aciduria, a defect of metabolite repair.** *J Inherit Metab Dis* 2007, **30**:681–689.

4. Fan J, Teng X, Liu L, Mattaini KR, Looper RE, Vander Heiden MG, Rabinowitz JD: **Human phosphoglycerate dehydrogenase produces the oncometabolite d-2-hydroxyglutarate**. *ACS Chem Biol* 2015, **10**:510–516.

5. Matsunaga H, Futakuchi-Tsuchida A, Takahashi M, Ishikawa T, Tsuji M, Ando O: **IDH1 and IDH2 have critical roles in 2-hydroxyglutarate production in D-2-hydroxyglutarate dehydrogenase depleted cells**. *Biochem Biophys Res Commun* 2012, **423**:553–556.

6. Linster CL, Noël G, Stroobant V, Vertommen D, Vincent M-F, Bommer GT, Veiga-da-Cunha M, Van Schaftingen E: **Ethylmalonyl-CoA decarboxylase, a new enzyme involved in metabolite proofreading**. *J Biol Chem* 2011, **286**:42992–43003.

7. Fujii T, Mukaihara M, Agematu H, Tsunekawa H: **Biotransformation of L-lysine to L-pipecolic acid catalyzed by L-lysine 6-aminotransferase and pyrroline-5-carboxylate reductase**. *Biosci Biotechnol Biochem* 2002, **66**:622–627.

8. Ning B, Elbein AD: **Cloning, expression and characterization of the pig liver GDP-mannose pyrophosphorylase**. *Eur J Biochem* 2000, **267**:6866–6874.

9. Ferguson F, McLennan AG, Urbaniak MD, Jones NJ, Copeland NA: **Re-evaluation of diadenosine tetraphosphate (Ap4A) from a stress metabolite to bona fide secondary messenger**. *Front Mol Biosci* 2020, **7**.

10. Veiga-da-Cunha M, Chevalier N, Stephenne X, Defour JP, Paczia N, Ferster A, Achouri Y, Dewulf JP, Linster CL, Bommer GT, et al.: **Failure to eliminate a phosphorylated glucose analog leads to neutropenia in patients with G6PT and G6PC3 deficiency**. *Proc Natl Acad Sci U S A* 2019, **116**:1241–1250.

11. Richard JP: **Kinetic parameters for the elimination reaction catalyzed by triosephosphate isomerase and an estimation of the reaction’s physiological significance**. *Biochemistry* 1991, **30**:4581–4585.

12. Ishii Y, Hashimoto T, Minakami S, Yoshikawa H: **The formation of erythronic acid 4-phosphate from erythrose 4-phosphate by glyceraldehyde-3-phosphate dehydrogenase**. *J Biochem* 1964, **56**:111–112.

13. Collard F, Baldin F, Gerin I, Bolsée J, Noël G, Graff J, Veiga-da-Cunha M, Stroobant V, Vertommen D, Houddane A, et al.: **A conserved phosphatase destroys toxic glycolytic side products in mammals and yeast**. *Nat Chem Biol* 2016, **12**:601–607.

14. Ash DE, Goodhart PJ, Reed GH: **ATP-dependent phosphorylation of α-substituted carboxylic acids catalyzed by pyruvate kinase**. *Arch Biochem Biophys* 1984, **228**:31–40.

15. Leblond DJ, Robinson JL: **Secondary kinase reactions catalyzed by yeast pyruvate kinase**. *Biochim Biophys Acta - Enzymol* 1976, **438**:108–118.

16. Bowes G, Ogren WL, Hageman RH: **Phosphoglycolate production catalyzed by ribulose diphosphate carboxylase**. *Biochem Biophys Res Commun* 1971, **45**:716–722.

17. Gerin I, Bury M, Baldin F, Graff J, Schaftingen E Van, Bommer GT: **Phosphoglycolate has profound metabolic effects but most likely no role in a metabolic DNA response in cancer cell lines**. *Biochem J* 2019, **476**:629–643.

18. Peracchi A, Veiga-da-Cunha M, Kuhara T, Ellens KW, Paczia N, Stroobant V, Seliga AK, Marlaire S, Jaisson S, Bommer GT, et al.: **Nit1 is a metabolite repair enzyme that hydrolyzes deaminated glutathione**. *Proc Natl Acad Sci* 2017, **114**:E3233–E3242.

19. Poore RE, Hurst CH, Assimos DG, Holmes RP: **Pathways of hepatic oxalate synthesis and their regulation**. *Am J Physiol Physiol* 1997, **272**:C289–C294.
